# Development and Validation of a Real-Time One-Pot RPA-CRISPR/Cas-Based Assay for Detection of ‘*Candidatus* Liberibacter Asiaticus,’ the Agent Associated with Citrus Greening Disease or Huanglongbing (HLB)

**DOI:** 10.3390/ijms27198728

**Published:** 2026-09-29

**Authors:** Brijesh B. Karakkat, Jarred Yasuhara-Bell

**Affiliations:** 1 Department of Entomology and Plant Pathology, North Carolina State University, Raleigh, NC 27607, USA; brijesh.karakkat@usda.gov; 2 Plant Pathogen Confirmatory Diagnostics Laboratory (PPCDL), Science and Technology (S&T), Plant Protection and Quarantine (PPQ), Animal and Plant Health Inspection Service (APHIS), United States Department of Agriculture (USDA), Laurel, MD 20708, USA

**Keywords:** *Candidatus* Liberibacter asiaticus, citrus greening, huanglongbing, RPA, CRISPR, one-pot, detection

## Abstract

Huanglongbing (aka: citrus greening) associated with ‘*Candidatus* Liberibacter asiaticus’ (CLas) is one of the most impactful citrus diseases throughout the world. The aim of this project was to support the sensitive and rapid detection of CLas to prevent the continued spread and destruction of US citrus crops by this psyllid vector-borne disease. Two multicopy targets specific to CLas were selected for assay development: the five-copy *nrdB* (ribonucleotide reductase-RNR) gene and a four-copy locus *4cp* (4CP). Published RNR RPA primers were used to develop a one-pot RPA-CRISPR assay, and RPA primers for 4CP were designed. crRNAs for both RNR- and 4CP-RPA-CRISPR/Cas assays were designed, evaluated through preliminary testing, and the best candidate primers and crRNA combinations were selected. The 4CP assay was selected and subjected to a battery of validation tests and determined fit-for-purpose. A field extraction protocol was also developed using commercial reagents, syringes and filters to bring kit sensitivity to the field. When combined with the assay, the complete workflow was able to detect CLas in infected citrus, down to a single infected leaf per subsample; the field workflow showed similar sensitivity to the laboratory-based workflow. While the extraction and assay proved useful, particularly for symptomatic leaves, its performance with asymptomatic leaves for early detection was not evaluated. The complexity of the assay in its current form hinders widespread practical applicability. In the future, revalidation of lyophilized reagents in a convenient kit can be performed for practical deployment; preliminary testing has shown success.

## 1. Introduction

The United States Department of Agriculture (USDA) Animal and Plant Health Inspection Service (APHIS) Plant Protection and Quarantine (PPQ) has engaged in several emergency programs to prevent and mitigate the effects of exotic plant pathogens in the US. One such pathogen, ‘*Candidatus* Liberibacter asiaticus’ (CLas), which is associated with huanglongbing (HLB) (AKA: citrus greening disease), is one of the most destructive diseases of citrus worldwide [1]. It has cost the Federal government and the citrus industry billions of dollars due to operational costs of survey and surveillance programs and lost economic activity, respectively. Despite a concerted effort, rapid, reliable, and sensitive detection assays for CLas that can be used in the field have been limited.

Huanglongbing is associated with an insect-vectored fastidious prokaryote. The bacterium resides in the sieve tube elements (phloem tissue) of infected plants and multiple organs (e.g., alimentary canal and salivary glands) of infected insects, namely psyllids. In nature, HLB exists in three forms that differ by a combination of environmental conditions and insect vectors. ‘*Candidatus* Liberibacter asiaticus’ (CLas), vectored by *Diaphorina citri*, is associated with a heat-tolerant form of HLB found in Asia [2]. Disease associated with ‘*Ca*. L. americanus’ (Lam) and ‘*Ca*. L. africanus’ (CLaf), vectored by *D. citri* and *Trioza erytreae,* respectively, are associated with heat-sensitive forms found in Brazil and southern Africa, respectively [2,3]. ‘*Candidatus* Liberibacter asiaticus’ is the most concerning due to its aggressive nature and heat tolerance and is designated a quarantine pathogen of regulatory concern in the US.

There are no cures for this devastating disease, and no commercial citrus cultivars displaying resistance are available [1,4,5]. Currently, disease management practices involve controlling the vector and destroying diseased trees to prevent spreading to healthy trees [6]. Identifying trees infected with CLas is a critical step in the disease management process. Sensitive detection is required at the early stages of disease presentation due to the low titer of CLas within the host, making PCR the gold standard for molecular diagnostics of this pathogen [7]. However, due to the low titer and uneven distribution of CLas within the plant, PCR assays can produce false negative or inconsistent results [1,8,9]. Thus, accurate detection requires a combination of an effective sampling strategy and a sensitive diagnostic. Visual assessment of disease symptoms in trees is the simplest initial screening method to suspect CLas infection and allows for targeted sampling to increase the likelihood of detection.

While PCR methods are sensitive and specific, they require equipment and processes restricted to a laboratory setting. Thus, trees are sampled in the field, and then samples must be sent to a laboratory for testing, which has a lag time that could result in decreases in sample integrity. At the laboratory, samples are typically extracted by a commercial kit, which requires time and skilled personnel. For delimitation surveys, positive results would then prompt a trip back to the field for resampling, and the process then repeats itself, requiring additional time for each successive find.

Field deployable diagnostics are advantageous because they allow on-site detection in real-time. Real-time surveillance can be accomplished based on results from a rapid field test, and many tools for this purpose have been developed that utilize loop-mediated amplification (LAMP) [10,11,12,13,14,15,16,17] and recombinase-polymerase amplification (RPA) technologies [4,5,18,19,20,21,22,23]. There also exists a commercial kit for CLas diagnostics, the AmplifyRP^®^ Acceler8^®^ for Las (Agdia Inc., Elkhart, IN, USA). Unfortunately, many of these assays were not developed and/or validated for practical applicability and thus are not readily utilized.

A new technology that has been rising in popularity in recent years is the Clustered Regularly Interspaced Short Palindromic Repeats (CRISPR)/CRISPR-associated (Cas) technology. While widely used as a gene editing technology, it has been applied to molecular diagnostics [24,25,26,27,28] and used to develop assays for the HLB pathogen [23,29,30]. Based on the claims within the literature regarding the specificity, sensitivity and utility of CRISPR-based diagnostics, the PPQ Citrus Health Response Program (CHRP) wanted to assess the benefits of this technology for field-deployable diagnostics. The aim of this project was to fulfill the need by developing and validating an RPA-CRISPR/Cas-based assay for detection of CLas, as well as a complementary field extraction protocol that will retain the sensitivity of a commercial kit for DNA extraction in the field.

## 2. Results

### 2.1. Assay Sensitivity

The limit of detection (LoD) was determined using serial dilutions of CLas-positive sample DNA diluted in healthy/clean ACP and citrus DNA. Dilutions were tested with the RNR real-time PCR to establish relative C_t_ values and enable comparison of the RPA-CRISPR assays to real-time PCR assay data (Supplementary Tables S1 and S2). The LoDs for 4CP and RNR RPA-CRISPR assays were the same for all three dilution series and across both platforms (AmpliFire^®^ Isothermal Fluorometer [Agdia Inc.] and Genie^®^ III [OptiGene Ltd., West Sussex, UK]), based on 100% positivity from three replicate wells; C_t_ value ~35, corresponding to ~5–10 copies of 4CP/rxn, or ~2–3 GECs/rxn. The 4CP RPA-CRISPR curves were a little faster and steeper than the RNR RPA-CRISPR curves at the LoD, allowing easier resolution of true amplification curves from negative samples; thus, the 4CP RPA-CRISPR assay was selected for full validation. The RPA-CRISPR assays appear 10-fold less sensitive than the RNR real-time PCR assay; however, this assay is intended for testing symptomatic leaves in the field.

The published real-time RNR RPA assay [18] was also evaluated for comparative sensitivity. When tested using the third dilution series (CLas-positive sample 2024-05 diluted in healthy citrus DNA), the LoD for this assay was similar to the RPA-CRISPR assays (Supplementary Tables S1 and S2); however, very weak “positive” amplification curves at the LoD were observed relative to the no-template control (NTC), making result interpretation more subjective. Only the Dilutions 5 and 6 (LoD and LoD^−1^) were evaluated with this assay for the CA and TX sample dilution series. Negative results were observed for this assay when tested in the AmpliFire^®^ Isothermal Fluorometer (Supplementary Table S1). When tested in the Genie^®^ III, both dilutions and the NTC produced positive results (Supplementary Table S2) due to the set threshold (protocol from this study) and curve drift in the NTC (Supplementary Figure S1). The differences in final relative fluorescence units (RFU) between amplification curves for the dilutions and the NTC were minimal. When disregarding the threshold established for the RPA-CRISPR assays, resolving true amplification curves from negative samples becomes highly subjective; thus, the sensitivity of the real-time RNR RPA assay was deemed lower than that of the RPA-CRISPR assays.

### 2.2. Assay Specificity

The assay demonstrated 100% specificity, confirming the target specificity reported previously [31]. The 4CP RPA-CRISPR assay produced positive results for all known CLas-positive ACP and citrus samples (Supplementary Table S3), demonstrating 100% inclusivity. The 4CP RPA-CRISPR assay produced negative results for all non-CLas pathogens of citrus (Table 1), demonstrating 100% exclusivity. One *Xanthomonas citri* pv. *citri* A^w^ pathotype sample (202403-01) tested positive by the 4CP RPA-CRISPR assay but also tested positive by the RNR PCR assay (Table 1). This sample came from an area where both pathogens are known to occur. Two different assays targeting two different CLas-specific markers produced positive results, confirming the presence of the pathogen and reinforcing the specificity and sensitivity of the assay. Positive results were not observed for any healthy citrus and ACP samples (100% selectivity).

### 2.3. Diagnostic Sensitivity and Specificity

Thirty-eight RNR real-time PCR-positive samples were tested, including eight low-titer (C_t_ ≥ 36) samples (Supplementary Table S4); these samples were not expected to produce positive results based on the assay LoD of C_t_ = ~35. Unexpectedly, three of these low-titer samples were able to produce positive results on both instruments, while another sample was only able to produce positive results on the Genie^®^ III. The diagnostic sensitivity was calculated as ~88% when including all PCR-positive samples tested. The low-titer samples were from 2018, and DNA degradation could be responsible for the negative results. However, when considering the LoD of the assay, all samples with C_t_ ≤ 35 produced positive results, demonstrating 100% diagnostic sensitivity. Negative results were obtained for all PCR-negative samples, demonstrating 100% diagnostic specificity.

To further assess the diagnostic sensitivity and applicability of the test workflow, known CLas-infected trees were sampled, and DNA was extracted using a commercial kit and the field extraction protocol. DNA was tested with the 4CP RPA-CRISPR and RNR PCR assays (Table 2). Positive results were produced for both assays with both extractions, when using symptomatic leaves, demonstrating 100% diagnostic sensitivity; C_t_ values were similar among samples. Directly comparing C_t_ values between kit extractions was difficult because each extraction represented a unique sample due to the uneven distribution of CLas within the plant. To account for this, a large composite sample was created and tested (N = 4 per extraction). Results revealed that the C_t_ values for both extractions were highly similar.

To simulate low-titer samples, the inclusion of only one infected midrib per extraction was tested. Three different known CLas-positive trees were tested: HLB22-06 (N = 4), B430 (N = 2) and B239 (N = 2). All four field extractions of HLB22-06 produced positive results with both assays and produced similar C_t_ values (~30–33), suggesting repeatable recovery (Table 2). In contrast, the commercial kit extraction produced positive results for only two of the four extractions; samples produced similar C_t_ values (~29–30). Both field extractions of B430 and B239 produced positive results with both assays (Table 2); C_t_ values were more variable with these samples, but this is likely due to sample differences. The commercial kit was not tested with B430 and B239 due to reduced performance with HLB22-06.

### 2.4. Assay Precision

Symptomatic leaves from a known CLas-infected plant were provided to three operators who then performed the field extraction and 4CP RPA-CRISPR protocols to determine if they could obtain positive results. All operators were able to obtain positive results with both the AmpliFire^®^ Isothermal Fluorometer and Genie^®^ III. All controls produced the expected results; the NTC produced negative results, and the synthetic positive control produced positive results for all operators after rounds of freeze-thaw. These results demonstrate acceptable intermediate precision. Results are binary, and each sample could have had a different titer; thus, no direct comparison could be made that would allow calculation of variance to quantify precision.

## 3. Discussion

The availability of validated diagnostic tools is the cornerstone of successful surveillance, quarantine, and eradication measures. A literature search was conducted and revealed several published assays described as being field-applicable, using technologies such as LAMP [10,11,12,13,14,15,16,17], RPA [4,5,18,19,20,21,22] and RPA-CRISPR [23,29,30]. However, many of these assays are not fit-for-purpose. Some of these publications provided extraction methods that are relegated to a laboratory setting, requiring commercial kits, centrifugation, liquid nitrogen and/or lyophilization [4,5,11,12,13,14,17,23]. Some of the published methods utilized crude field-applicable extraction protocols [5,10,15,16,19,21,22,23,29]; however, these methods use reduced amounts of tissue inputs and/or diluted samples in buffers relative to the 200 µL DNA elution produced by commercial kits, decreasing the pathogen input into each reaction and thereby reducing the overall sensitivity of these tests. The AmplifyRP^®^ Acceler8^®^ for Las (Agdia Inc.) also has a reduced sample input and requires sample dilution, and it is relatively expensive; list price without tax or shipping is $35 USD per reaction. Some of the published assays utilize lateral flow assessment of results, which requires opening reaction tubes and risks lab-wide/site-wide contamination [4,5,13,18,22]. The assays by Li et al. [29] and Wheatley et al. [30] are two-pot assays requiring opening RPA reaction tubes and transferring amplicons to the CRISPR reaction, thus risking contamination. These assays also have increased reaction times, as two separate reactions must be run before result analysis.

Many of these assays were not directly compared to the most sensitive gold-standard PCR assay [32]. Molecular diagnostics for CLas suffer challenges due to the uneven distribution within the plant and potential for low titers, even with PCR. Sampling protocols have been developed to increase the likelihood of detection by sampling multiple leaves from different parts of the plant. By reducing the tissue input, crude extraction methods reduce sample representation from a single or multiple trees. Dilution of crude samples to ensure assay compatibility reduces overall test sensitivity compared to PCR tested using kit-extracted DNA eluted into a 100–200 µL volume. Isothermal technologies can come close to matching the sensitivity of PCR but are generally less sensitive. When the lower sensitivity inherent in the assay is compounded by the decrease in sensitivity attributed to the extraction process, the chances of false negative results increase and preclude applicability in the field. The aim of this project was to develop and validate a portable assay for specific and sensitive detection of CLas in the field, as well as a field extraction protocol that can mimic commercial kit sensitivity in the field.

An RPA-CRISPR assay was developed to detect the four-copy 4CP gene of CLas. This assay performed better than a similar assay designed to detect the five-copy RNR gene, as well as a published real-time RNR RPA assay [18]. Validation testing demonstrated 100% specificity; however, there was a loss in sensitivity compared to PCR. Based on the inclusion of low-titer diagnostic samples below the assay’s LoD, the calculated diagnostic sensitivity was 88.37%. However, this value should be taken with a grain of salt, as results could be biased by inclusion of additional higher-titer samples, or reduction in the number of low-titer samples. For example, if the known-positive ACP samples (N = 31) are included from specificity testing, the new calculated diagnostic sensitivity is 93.75%. Additionally, 100% diagnostic sensitivity was observed when testing CLas-infected citrus and comparing results with those from commercial kit extractions and PCR.

An extraction method was designed to accompany the test in the field. When combined, the test was able to detect CLas in symptomatic citrus. Due to the assay’s reduced sensitivity, it is possible that low-titer samples would produce negatives. The protocol is intended for testing symptomatic tissues, so this is not expected to be a problem. There may be an issue if the assay is used for “early detection” (presymptomatic), which was not evaluated in this study. Attempts to replicate low-titer situations were made; however, it was very difficult to replicate low-titer scenarios with this host–pathogen system. To account for sampling strategies, one infected midrib among healthy midribs was tested by lab-based and field protocols. Results showed that the field test could reliably detect one infected midrib, based on the experimental parameters; the titer of the single midrib input was relatively high, and it is unknown how the test will perform in other circumstances. Results also showed that the field test outperformed the lab-based test, as one of four replicates produced negative results and another produced high C_t_ values; all produced equivalent COX (plant) C_t_ values. This is believed to be due to the increased tissue input for the field extraction, which may increase the likelihood of recovering CLas. Therefore, while the assay itself does have reduced sensitivity relative to PCR, the overall field test appears equivalent to the lab-based test. As the assays by Morán et al. [21] and Fu et al. [19] appeared well designed and validated, it is possible to combine these assays with the field extraction protocol described here to prevent loss in sensitivity due to sample dilution.

Attempts were made to improve the RPA-CRISPR assays. The EnGen^®^ Lba Cas12a (Cpf1) enzyme (New England Biolabs, Ipswich, MA) comes with a 10× buffer (NEBuffer™ r2.1). Xu et al. [28] suggested that switching NEBuffer™ r2.1 to NEBuffer™ 4 (New England Biolabs) could drastically improve results. This was explored, but the NEBuffer™ r2.1 appeared to produce better results with these assays. Multiple crRNAs were designed for both the 4CP and RNR RPA-CRISPR assays. The use of two crRNAs for each RPA-CRISPR assay was evaluated, as well as a dual RPA assay combining the 4CP and RNR assays into a single reaction. Results suggested that the use of two crRNAs or dual RPA did not improve assay kinetics. The field extraction protocol was the result of many rounds of testing and optimization. Attempts were made to apply the extraction method to psyllids, but the extraction method failed to recover CLas-infected psyllid DNA spiked into extractions; the reason for this was not explored following multiple retests and attempts at troubleshooting.

The cost per reaction of the 4CP RPA-CRISPR assay was calculated to be ~$3.22 USD, based on the reagents and oligonucleotides used per reaction, plus a 20% increase to account for disposable supplies like tips, tubes, and gloves; this does not reflect operational costs. The cost per reaction of the published real-time RNR RPA assay was calculated to be ~$9.76 USD, with substantial cost coming from the AmplifyRP^®^ XRT Discovery Kit (TwistDx™ Ltd., Cambridge, UK). Though not validated for use with TwistDx™ reagents, the cost of the published assay using the same reagents as the RPA-CRISPR assay was calculated to be ~$3.60 USD. Regardless, the 4CP RPA-CRISPR assay appears to be cheaper than the published RPA in any form.

The cost per citrus sample when using the field extraction was calculated to be ~$10.32 USD, based on the use of a specific brand and number of filters and syringes; use of a different brand and/or number of syringes and filters could increase costs. The calculated cost does not reflect the operational costs to perform the extractions, nor does it include the one-time cost of the instrument to help dry the membranes; the WOLFBOX MF50 Electric Air Duster was $39.99 each, and it may be desirable to purchase multiple. When compared to the commercial kit extraction, the field extraction is basically the same cost per sample; however, it produces more waste. The commercial kit extraction protocol requires the DNeasy^®^ Plant Mini Kit (Qiagen, Germantown, MD, USA), lysing matrix A tubes and an extra ceramic bead. The cost of the commercial kit extraction was calculated to be ~$10–$11 per sample, depending on the size of the kit (50 preps vs. 250 preps) and number of lysing matrix A tubes and beads purchased (50, 100 or 500). This also does not reflect the operational costs to perform the extraction, nor does it include the one-time cost of the microcentrifuge, thermomixer, and FastPrep-24™ homogenizer; it also does not reflect the cost of 100% molecular-grade ethanol for reagent preparation.

The cost of using the synthetic gBlocks^TM^ positive control was also calculated based on rehydration and dilution to 100 ag/µL in 0.5 mg/mL poly(A) in TE buffer. The cost for every 1000 reactions was calculated to be $8.27 USD for each control; the largest contributing factor to cost was the poly(A), and then the TE buffer. This equates to less than 1¢ per reaction per control; cost calculations do not reflect operational costs.

## 4. Materials and Methods

### 4.1. Samples and DNA Extraction

Leaf samples included CLas-infected *Citrus* spp. maintained at PPCDL, CLas-infected *Citrus* spp. maintained at the Exotic Pathogens of Citrus Collection (EPCC) at USDA Agricultural Research Service (ARS) (Beltsville, MD, USA), and various CLas-infected *Citrus* spp. collected as diagnostic and/or survey samples from multiple states. Asian citrus psyllid (ACP) samples included CLas-infected *Diaphorina citri* collected as diagnostic and/or survey samples from multiple states. Various pathogens of citrus were also included in this study (Table 1); samples included infected plant material and bacterial and fungal cultures.

DNA was extracted from leaf midrib tissue using the DNeasy^®^ Plant Mini Kit (Qiagen, Germantown, MD, USA) with minor modifications. Briefly, midribs with petioles were removed from 12 individual leaves and chopped into small pieces (~1–2 mm in length). Samples were mixed well to create a uniform composite sample from which 200 mg of chopped tissue were processed with 800 µL AP1 buffer and 8 µL RNase A (100 mg/mL) in a Lysing Matrix A tube with an additional ceramic bead (MP Biomedical, Irvine, CA, USA), using a FastPrep-24™ (MP Biomedical), for 2 runs at speed setting 6.0 for 40 s each. Tubes were incubated in a thermomixer at 65 °C for 10 min, with 500 rpm agitation. Following incubation, 260 µL Buffer P3 was added to each tube, followed by vortexing and incubation on ice for at least 5 min. The remaining steps of the extraction were carried out according to the manufacturer’s instructions. Citrus canker samples were extracted according to Yasuhara-Bell et al. [33].

DNA was extracted from ACPs using the DNeasy^®^ Blood & Tissue Kit (Qiagen) with modifications. Briefly, 1–25 adults or 1–75 immatures were added to Lysing Matrix A tubes containing 360 µL PBS (phosphate-buffered saline–50 mM potassium phosphate, 150 mM NaCl, pH 7.2) and then processed using a FastPrep-24™ run at speed setting 6.0 for 40 s. Tubes were centrifuged for 10 min at 14,000 rpm, and then 180 µL supernatant was transferred to a new 1.5 mL microcentrifuge tube. Lysate was mixed with 20 μL proteinase K and 200 μL Buffer AL (without added ethanol) and then incubated at 56 °C for 10 min. Following incubation, 200 μL molecular-grade ethanol (200 proof) was added to each tube. The AW1 and AW2 steps were as per the manufacturer’s instructions, except the column was centrifuged for 1 min at 14,000 rpm, moved to a new 1.5 mL microcentrifuge tube, and centrifuged again for 2 min at 14,000 rpm for the AW2 wash step. DNA was eluted twice using 50 μL Buffer AE each time, following a 1 min incubation at room temperature and then centrifugation at >8000 rpm. DNA was extracted from bacterial and fungal cultures using the DNeasy^®^ Blood & Tissue Kit, according to the manufacturer’s instructions.

### 4.2. Field DNA Extraction

A field extraction protocol was developed in this study to accompany the portable assay. The protocol incorporates syringes and filters in combination with buffers AW1 and AW2 (Qiagen) to bring a kit extraction to the field. Midribs with petioles were removed from 20 individual leaves, and a ~2 cm section from the petiole ends was removed and placed into a mesh sample bag (Agdia Inc., Elkhart, ID, USA) containing 3 mL CTAB Extraction Buffer (OPS Diagnostics, Lebanon, NJ, USA). Samples were pulverized on a hard flat surface using a hard blunt object (e.g., large ceramic pestle or hammer), and then samples were placed upright in a stand to allow bubbles/foam to dissipate and the liquid to pool at the bottom of the bag (2–5 min). Lysate was poured into a container (e.g., weighing boat), aspirated into a 10 mL Luer Lock syringe, and then plunged through a 13 mm glass fiber VWR^®^ Syringe Filter (1.0 µm) (Avantor, Inc., Radnor Township, PA, USA) prefilter. Approximately 10 mL of air was plunged twice to expel as much lysate as possible; this step was done following each liquid plunge through a filter. Flow-through was mixed with 3 mL AW1, aspirated, and then plunged through two new 13 mm glass fiber VWR^®^ syringe filters (1.0 µm) to capture DNA. The two filters were washed separately by plunging ~2 mL AW1 and then ~2 mL 100% molecular-grade ethanol (200 proof) through each filter. Filters were dried using a handheld portable blower capable of ≥60,000 rpm (e.g., MegaFlow 50, Wolfbox, Denver, CO, USA). At 110,000 rpm and an ambient temperature of ~20 °C, 5 min was sufficient to dry the membrane; increased ambient temperature and decreased relative humidity can expedite this process. To elute DNA, 0.2 mL 1× TE buffer (Promega, Madison, WI, USA) was aspirated into a 1 mL Luer Lock syringe and then plunged through the two capture filters in reverse order of capture. A graphical schematic of the workflow is shown in Supplementary Figure S2.

### 4.3. Primer Design

The genome sequence of ‘*Candidatus* Liberibacter asiaticus’ strain A4 (Accession: CP010804) was downloaded from NCBI GenBank and *4cp* (regions 3538-4764, 885129-886355, 909333-910559 and 1000011-1001237) and *nrdB* (ribonucleotide reductase-RNR) (regions 4924-5301, 67911-68970, 808778-809836, 955199-956257 and 999474-999851) sequences were extracted. The 4CP sequences were analyzed using PrimedRPA v1.0.3 [34] for RPA primer design using default parameters. Published RPA primers targeting RNR were used in this study [18]. Primer binding sites were mapped to target sequences in Geneious Prime^®^ 2025.0.2 [35], and the target amplicons were extracted and rescreened using NCBI BLASTn [36] (megablast and blastn with default parameters) to assess matches to non-targets; whole-genome shotgun contigs (wgs), core nucleotide (core_nt), Refseq Genome (refseq_genomes), and Refseq Reference Genome (refseq_reference_genomes) databases were queried. Amplicon sequences for 4CP and RNR were used to design/select CRISPR guide RNA (crRNA) spacer sequences based on suboptimal protospacer adjacent motifs (PAM) [37] for one-pot compatibility. The primers and crRNA sequences used in this study are shown in Table 3.

### 4.4. RPA Reaction Conditions

The real-time RNR RPA assay [18] was performed as described in the original publication; see Table 3 for primer and probe sequences. The AmplifyRP^®^ XRT Discovery Kit (Agdia Inc.) was used, and assays were performed as 24 µL reactions containing 14.75 µL rehydration buffer, 1.05 µL each primer (10 µM each), 0.3 µL probe (10 µM), 3.35 µL molecular-grade water and 2 µL DNA template, per reaction pellet. Following preparation of the reaction pellet, 1.5 µL of magnesium acetate (280 mM) was added carefully to the caps of each tube. Tube caps were closed carefully before spinning the tubes down and then running reactions at 39 °C for 20 min in the Genie^®^ III (OptiGene Ltd., West Sussex, UK); this was done to ensure all reactions started at the same time for normalization and comparison purposes.

### 4.5. RPA-CRISPR/Cas Reaction Conditions

RPA-CRISPR/Cas assays were performed as 30 µL reactions. Each reaction consisted of two components (A and B). TwistAmp^®^ Basic lyophilized pellets (TwistDx™ Ltd., Cambridge, UK) were rehydrated with 44.5 µL Component B per pellet. Rehydrated pellets were split into two new reaction tubes (22 µL each), 2 µL DNA was added to each reaction, and then 6 µL Component A was added carefully to the caps of each tube. Tube caps were closed carefully before spinning the tubes down and then running reactions at 39 °C for 20 min, in both the AmpliFire^®^ Isothermal Fluorometer (Agdia Inc.) and Genie^®^ III; this was done to ensure all reactions started at the same time for normalization and comparison purposes. The compositions of Components A and B are shown in Supplementary Table S5.

For the AmpliFire^®^ Isothermal Fluorometer, reaction conditions and analysis parameters were programmed into QR codes using AmpliCode™ (Agdia Inc.) (https://amplicode.agdia.com/, access on 31 July 2026); the QR codes are scanned by the instrument to set up a run (Supplementary Figure S3). For the Genie^®^ III, the yellow channel and melt curve analysis were disabled, and analysis parameters were updated; Type: Threshold, Plot: Normalized, Range X 180 to 1200 s, and Range Y 1200 to 1200 F. Additionally, result output settings were changed such that the instrument would display “Positive” and “Negative” for corresponding results based on curve analysis, thus eliminating user interpretation.

### 4.6. PCR Reaction Conditions

A duplex real-time PCR assay targeting the *nrdB* (RNR) gene [32] of CLas and the *cox* gene [38] of the citrus (internal control) was used in this study to verify and quantify DNA extractions and dilutions; primers and probe sequences are shown in Table 3. Reactions contained 1X PerfeCTa^®^ qPCR ToughMix^®^ Low ROX™ (Quantabio, Beverly, MA, USA), 240 nM primers, 120 nM probe and 2 µL DNA sample in a final volume of 25 µL. Reactions were run in the QuantStudio™ 5 Real-Time PCR System (Applied Biosystems, Waltham, MA, USA) according to the following cycling conditions: initial denaturation at 95 °C for 25 s, followed by 40 cycles of 95 °C for 3 s and 60 °C for 40 s.

### 4.7. Synthetic Positive Control

A synthetic gBlocks™ (Integrated DNA Technologies, Coralville, IA, USA) positive control was designed for use with the described CLas 4CP RPA-CRISPR assay (Supplementary Table S6). The gBlocks™ was 250 bp, containing the 147 bp 4CP RPA amplicon, plus an additional 56 bp upstream and 47 bp downstream. The control was tested at ~100 ag/µL (~370 copies/µL) in 1× TE buffer with 0.5 mg/mL polyadenylic acid (poly(A)) (Roche Diagnostics, Mannheim, DE).

### 4.8. Assay Validation Testing

Validation testing was performed to evaluate the analytical specificity and sensitivity, diagnostic sensitivity and specificity, repeatability, and precision of the optimized assay. DNA from known CLas-positive citrus and ACP were used to determine analytical specificity (inclusivity) (Supplementary Table S3). DNA from other bacterial, viral and fungal pathogens of citrus were also used to determine analytical specificity (exclusivity) (Table 1). Additionally, DNA from healthy citrus was used to determine analytical specificity (selectivity). Forty-four diagnostic samples that tested positive (N = 38) or negative (N = 6) for CLas by PPCDL diagnosticians previously were used to determine diagnostic specificity and sensitivity (Supplementary Table S4).

Three different dilution series were used to establish assay sensitivity and define the limit of detection (LoD). Two well-characterized dilution series were provided by Z. Yan from PPCDL: CLas DNA from CA and TX samples diluted in clean ACP DNA. The third dilution series was established for this study using DNA from CLas-positive sample 2024-05 from Florida diluted in healthy citrus DNA. Dilutions series consisted of several 10-fold and half-log dilutions. Approximate copies per reaction were calculated based on previous data from an in-house validation of the PCR assay using dilutions of plasmids containing the RNR target.

Intermediate precision (N = 9) was evaluated using data from three operators from different laboratory teams at USDA APHIS PPQ S&T PPCDL. Symptomatic leaves were collected from the same CLas-positive tree and provided to participants, along with a copy of the testing protocol. Samples were processed using the field extraction protocol and then tested using the RPA-CRISPR protocol on both instruments. Samples were tested in triplicate, along with the synthetic positive control and a no-template control (NTC).

All assay results were binary, with positive results based on the presence of an appropriate amplification curve, while negative results were based on the absence of an amplification curve. Analysis parameters were programmed into the portable instruments, and result outputs were +/− or Positive/Negative for the AmpliFire^®^ Isothermal Fluorometer and Genie^®^ III, respectively; no interpretation was required.

## 5. Conclusions

The test workflow described here, which includes a field extraction and RPA-CRISPR assay based on the 4CP gene of CLas, was validated and shown to be fit-for-purpose. The assay demonstrated comparable specificity to PCR, and the test workflow showed comparable sensitivity to PCR. Additionally, results demonstrated that the field extraction was compatible with PCR assays, requiring no clean-up for use. Therefore, DNA samples producing positive results in the field can be sent to a laboratory for confirmatory testing, along with new plant sample material. This is beneficial due to the uneven distribution of CLas within the plant. While new tissue samples come from the same plant, they may produce vastly different results; thus, it is beneficial to be able to test the original extracted DNA sample. The assay presented here represents a useful diagnostic tool that can be deployed on-site to aid domestic survey and CLas detection efforts. However, in its current form, it requires mixing many reagents into two separate components in a laboratory setting, which are then stored separately and brought to the field for use, which reduces its practical applicability. Lyophilization of Components A and B separately and then mixing the two lyophilized reagents in a single reaction tube to create an easy-to-use “kit” has demonstrated success. In the future, revalidation of the assay in kit form can be conducted for practical deployment of the assay.

## Figures and Tables

**Table 1:** Non-CLas pathogens of citrus tested in this study and corresponding RNR real-time PCR and 4CP RPA-CRISPR data.

Sample	Organism	Type	Source ^a^	RNR PCR ^b^	4CP RPA-CRISPR
CLaf	‘*Candidatus* Liberibacter africanus’	Infected plant	Z. Yan	-	-
CLam	‘*Candidatus* Liberibacter americanus’	Infected plant	Z. Yan	-	-
Lcr 150210A	*Liberibacter crescens*	Culture	Z. Yan	-	-
Lcr 140180	*Liberibacter crescens*	Culture	Z. Yan	-	-
CVC M1	*Xylella fastidiosa* subsp. *pauca*	Infected plant	Z. Yan	-	-
202402-01A	*Xanthomonas citri* pv. *citri* A	Infected plant	PPCDL	-	-
202403-01 ^c^	*Xanthomonas citri* pv. *citri* A^W^	Infected plant	PPCDL	33.93	+
202501-01	*Xanthomonas citri* pv. *citri* A^W^	Infected plant	PPCDL	-	-
53	*Xanthomonas citri* pv. *citri* A^W^ (culture)	Culture	G. Santillana	-	-
WBDL	‘*Candidatus* Phytoplasma *aurantifolia*’	Infected plant	U of Bologna	-	-
2022-01 (B)	*Alternaria alternata*	Culture	PPCDL	-	-
2022-01 (C)	*Colletotrichum gloeosporioides*	Culture	PPCDL	-	-
2017-01 (C-5)	*Colletotrichum queenslandicum*	Culture	PPCDL	-	-
CYVCV-2-2	Citrus yellow vein clearing virus (CYVCV)	Infected plant	PPCDL	-	-
T#3-3	Citrus yellow vein clearing virus (CYVCV)	Infected plant	A. Roy	-	-
S#3, T#6	Citrus yellow vein clearing virus (CYVCV)	Infected plant	A. Roy	-	-
T#4-2	Citrus yellow vein clearing virus (CYVCV)	Infected plant	A. Roy	-	-
CTLV-2	Citrus tatter leaf virus (CTLV)	Infected plant	PPCDL	-	-
B332-2	Citrus chlorotic dwarf-associated virus (CCDaV)	Infected plant	A. Roy	-	-
B175	Citrus yellow mosaic virus (CYMV)	Infected plant	A. Roy	-	-
B27	Citrus tristeza virus (CTV)	Infected plant	A. Roy	-	-
GC 3-1	*Phyllosticta citricarpa*	Culture	K. Zeller	-	-
S4275C	*Elsinoë australis*	Culture	K. Zeller	-	-

^a^ A. Roy-USDA Agricultural Research Service (ARS); Beltsville, MD, Exotic Pathogens of Citrus Collection (EPCC); G. Santillana-USDA APHIS PPQ S&T Plant Pathogen Confirmatory Diagnostics Laboratory (PPCDL); K. Zeller–USDA APHIS PPQ S&T PPCDL; PPCDL—original sample was received for diagnostic testing by the USDA APHIS PPQ S&T PPCDL; U of Bologna—DNA purchased from A. Bertaccini, University of Bologna, Department of Plant Pathology, Bologna, Italy; Z. Yan—USDA APHIS PPQ S&T PPCDL. ^b^ RNR PCR using primers from Zheng et al. [32]. ^c^ Sample 202403-01 was collected from an area under federal quarantine for both citrus canker and HLB disease.

**Table 2:** Comparison of CLas-infected citrus samples processed by commercial kit and field extraction methods ^a^.

Sample ID	Host	DNeasy^®^ Plant Mini Kit (Qiagen, Germantown, MD, USA)		Field Extraction	
		RNR PCR	4CP-CRISPR	RNR PCR	4CP-CRISPR
1	*Citrus* sp.	31.33 ± 0.42	+	29.96 ± 0.85	+
2	*Citrus* sp.	31.03 ± 0.44	+	27.65 ± 0.06	+
HSG2-1	*Citrus sinensis*	18.90 ± 0.16	+	21.02 ± 0.99	+
HSG2-2	*Citrus sinensis*	19.42 ± 0.51	+	20.09 ± 0.23	+
HSG10-6-2	*Citrus sinensis*	22.27 ± 0.22	+	23.95 ± 0.09	+
HSG10-22-1	*Citrus sinensis*	21.12 ± 0.37	+	22.56 ± 0.03	+
HSG10-22-2	*Citrus sinensis*	21.55 ± 0.14	+	23.84 ± 0.18	+
HSG5-37-1	*Citrus hystrix*	20.37 ± 0.32	+	19.55 ± 0.04	+
HSG5-37-2	*Citrus hystrix*	16.91 ± 0.17	+	22.65 ± 0.37	+
HSG5-9	*Citrus sinensis*	19.48 ± 0.46	+	22.22 ± 0.04	+
Composite 1	*Citrus sinensis*	22.27 ± 0.13	+	24.18 ± 0.23	+
Composite 2	*Citrus sinensis*	23.13 ± 0.31	+	23.05 ± 0.07	+
Composite 3	*Citrus sinensis*	NT	NT	22.63 ± 0.26	+
Composite 4	*Citrus sinensis*	NT	NT	23.48 ± 0.24	+
HLB22-06-A ^b^	*Citrus sinensis*	30.06 ± 0.08	+	29.43 ± 0.27	+
HLB22-06-B ^b^	*Citrus sinensis*	-	-	32.47 ± 0.28	+
HLB22-06-C ^b^	*Citrus sinensis*	-	-	31.57 ± 0.09	+
HLB22-06-D ^b^	*Citrus sinensis*	29.06 ± 0.10	+	33.02 ± 0.01	+
B430-A ^c^	*Citrus sinensis*	NT	NT	26.06 ± 0.27	+
B430-B ^c^	*Citrus sinensis*	NT	NT	24.15 ± 0.11	+
B239-A ^c^	*Citrus sinensis*	NT	NT	25.12 ± 0.00	+
B239-B ^c^	*Citrus sinensis*	NT	NT	28.94 ± 0.01	+

^a^ NT = not tested, ^b^ Tests using one CLas-positive midrib among 11 healthy midribs. ^c^ Tests using one CLas-positive midrib among 20 healthy midribs.

**Table 3:** Oligonucleotide primers and probes used in this study ^a^.

Oligo	Sequence (5′→3′)	Target	Amplicon (bp)	Reference
RNR RPA F3	CCTCCTCGAAATCGCCTATGCACATGAAACAA	*nrdB*	170	[18]
RNR RPA R3	CTTCACTCATCCAAGGGAAAGGGTTTTCTGTA			
RNR RPAp XRT	TGAACAATATATGCAGTTCATCGCCAA[FAM]CG[THF]CG[BHQ]TGTCACCAAATC-C3			
crRNA-RNR ^b^	GCGATGAACTGCATATATTG			This Study
4CP-F	GGCTATAGGCATGACATTCTCGTCAGAATTAA	4CP	147	This Study
4CP-R	CGTCATAGAACTGGAAGAGATGCAATTACCAT			
crRNA-4CP ^b^	AAGTATATCGCGTATCTTGA			
RNR1-F	CATGCTCCATGAAGCTACCC	*nrdB*	81	[32]
RNR1-R	GGAGCATTTAACCCCACGAA			
RNR-P	FAM-CCTCGAAATCGCCTATGCAC-BHQ-1			
COXf	GTATGCCACGTCGCATTCCAGA	*cox*	68	[38]
COXr	GCCAAAACTGCTAAGGGCATTC			
COXp	JOE-ATCCAGATGCTTACGCTGG-BHQ-2			
ssDNA Reporter	FAM-CCCCCC-BHQ-1	N/A	N/A	[39]

^a^ Oligonucleotides used in this study were synthesized by Integrated DNA Technologies (IDT, Coralville, IA, USA). ^b^ The sequence represents the CRISPR spacer sequence only. The crRNA backbone sequence is determined by the enzyme used; only the “spacer” sequence needs to be specified when ordering.

## Data Availability

All relevant data are provided in the main document and Supplementary Materials.

## Supplementary Materials

The following supporting information can be downloaded at: https://www.mdpi.com/article/10.3390/ijms27198728/s1.

## Equal Opportunity/Non-Discrimination Statement

In accordance with Federal civil rights law and the U.S. Department of Agriculture (USDA) civil rights regulations and policies, the USDA, its Agencies, offices, and employees, and institutions participating in or administering USDA programs are prohibited from discriminating based on race, color, national origin, religion, sex, disability, age, marital status, family/parental status, income derived from a public assistance program, political beliefs, or reprisal or retaliation for prior civil rights activity, in any program or activity conducted or funded by USDA (not all bases apply to all programs). Remedies and complaint filing deadlines vary by program or incident. Persons with disabilities who require alternative means of communication for program information (e.g., Braille, large print, audiotape, American Sign Language, etc.) should contact the State or local Agency that administers the program or contact USDA through the Telecommunications Relay Service at 711 (voice and TTY). Additionally, program information may be made available in languages other than English. To file a program discrimination complaint, complete the USDA Program Discrimination Complaint Form, AD-3027, found online at How to File a Program Discrimination Complaint and at any USDA office or write a letter addressed to USDA and provide in the letter all of the information requested in the form. To request a copy of the complaint form, call (866) 632-9992. Submit your completed form or letter to USDA by: (1) mail: U.S. Department of Agriculture, Office of the Assistant Secretary for Civil Rights, 1400 Independence Avenue, SW, Mail Stop 9410, Washington, D.C. 20250-9410; (2) fax: (202) 690-7442; or (3) email: program.intake@usda.gov. USDA is an equal opportunity provider, employer, and lender.
