# Supplementary material for: Development and Validation of a Real-Time One-Pot RPA-CRISPR/Cas-Based Assay for Detection of ‘Candidatus Liberibacter Asiaticus,’ the Agent Associated with Citrus Greening Disease or Huanglongbing (HLB)
Source: Int J Mol Sci. 2026 Sep 29;27(19):8728. doi: 10.3390/ijms27198728 (PMC13664829; doi:10.3390/ijms27198728)
Supplement: Supplementary file 1 [file ijms-27-08728-s001.zip › ijms-4569600-supplementary.pdf]

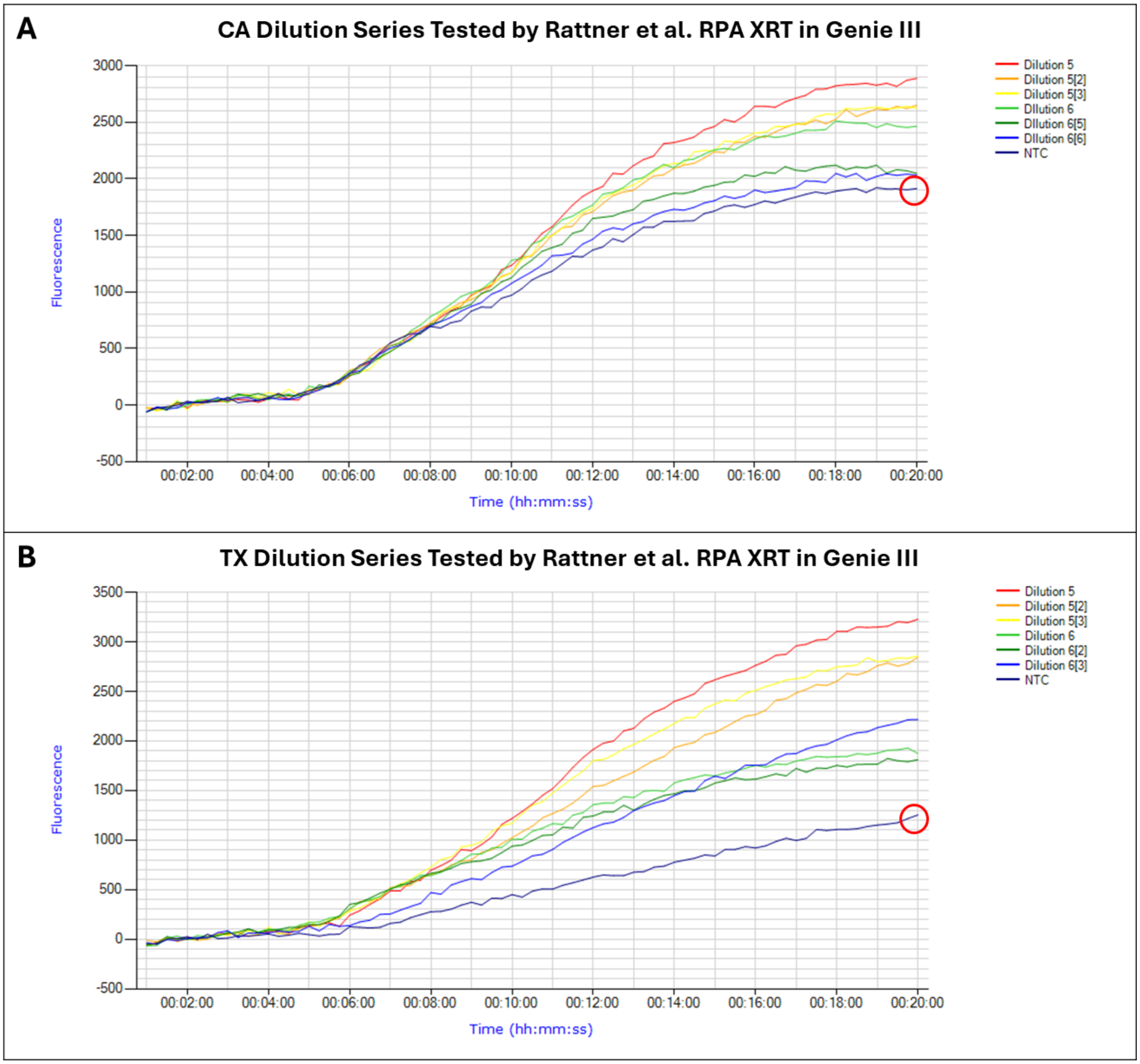

Figure S1. Amplification curves for the published RNR RPA XRT by Rattner et al. (2022) for two CLas dilutions in ACP background using the Genie® III. Dilutions 5 (LoD of RPA-CRISPR) and 6 of CLas in ACP background from **A)** CA and **B)** TX were tested with the published RNR RPA XRT. Amplification plots show that the NTC also has increased RFU by the end of the run. The RFU of the NTC (circled in red) varied between runs, ~1,000-2,000 RFU. Based on results, there is only a 1,000-2,000 RFU difference between Dilution 5 and the NTC. The publication didn't set any analysis parameters (e.g.: threshold values) for positive result determination. Therefore, looking at the curves for comparison to determine positive versus negative results is highly subjective. When using the parameters set for the RPA-CRIPSR/Cas-based assay, all wells returned "positive" results due to all curves crossing the set threshold.

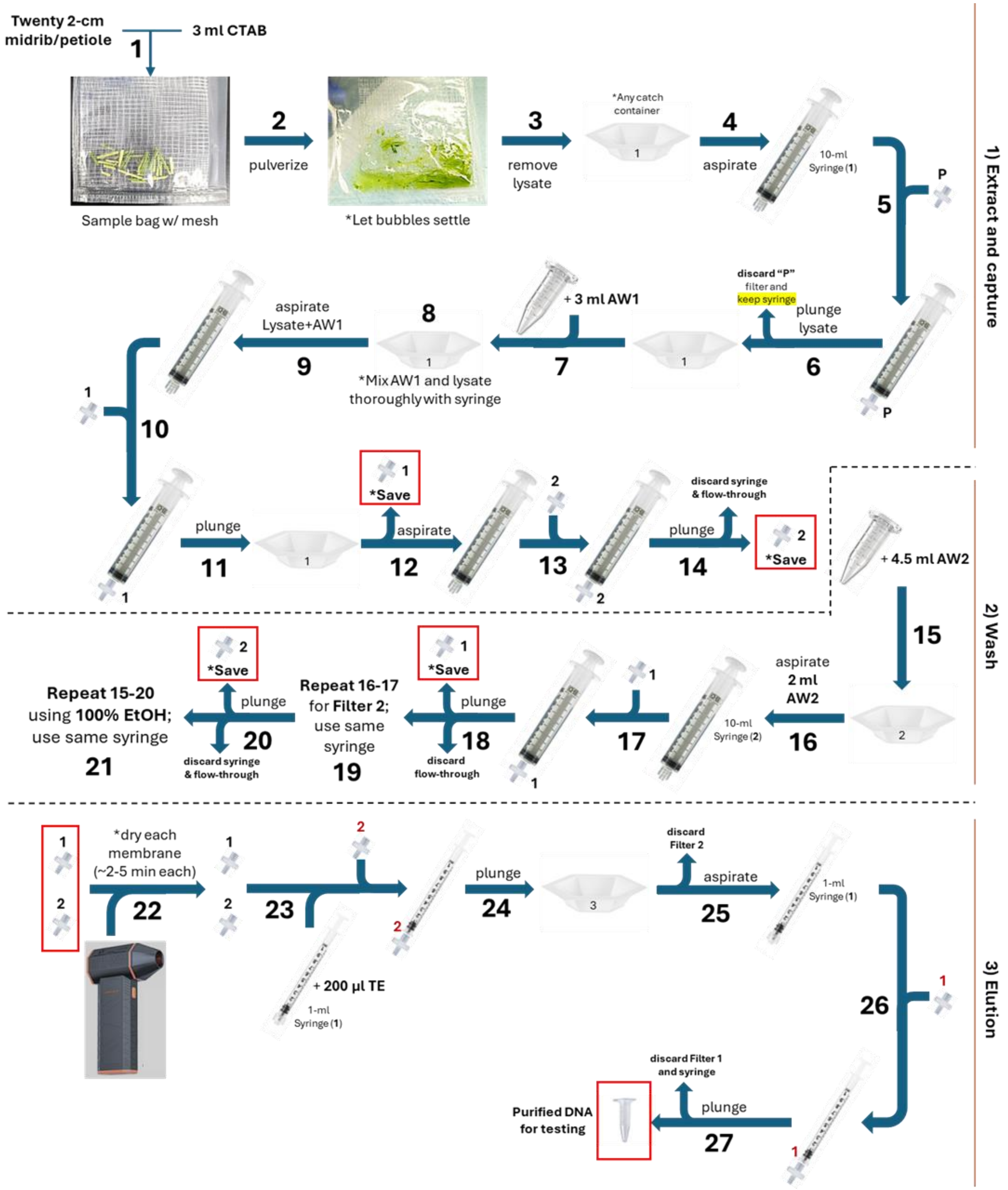

Figure S2. Graphical schematic showing the field extraction workflow.

Figure S3. AmpliFire® Isothermal Fluorometer reaction QR code. The QR code is scanned by the AmpliFire® Isothermal Fluorometer and automatically loads the reaction conditions and analysis parameters. Result outputs are binary (+/-).

Table S1. Sensitivity data using dilution series tested in the AmpliFire® Isothermal Fluorometer.

			California											
			4CP-RPA CRISPR				RNR-RPA CRISPR				RNR-XRT-RPA			
**Dilution**	**RNR C_t**	**~4CP Copies**	**Rep 1**	**Rep 2**	**Rep 3**	**%Positive**	**Rep 1**	**Rep 2**	**Rep 3**	**%Positive**	**Rep 1**	**Rep 2**	**Rep 3**	**%Positive**
**1**	24.47	13600.00	+	+	+	100.00%	NT	NT	NT	-	NT	NT	NT	-
**2**	27.74	1360.00	+	+	+	100.00%	NT	NT	NT	-	NT	NT	NT	-
**3**	31.02	136.00	+	+	+	100.00%	NT	NT	NT	-	NT	NT	NT	-
**4**	32.69	44.80	+	+	+	100.00%	NT	NT	NT	-	NT	NT	NT	-
**5**	33.95	13.60	+	+	+	100.00%	+	+	+	100.00%	-	-	-	0.00%
**6**	36.05	4.53	+	-	-	33.33%	+	-	-	33.33%	-	-	-	0.00%
**7**	37.47	1.36	+	-	+	66.67%	NT	NT	NT	-	NT	NT	NT	-
**8**	37.95	0.45	-	-	-	0.00%	NT	NT	NT	-	NT	NT	NT	-
**NTC**	-	-	-	-	-	-	-	-	-	-	-	-	-	-

			Texas											
			4CP-RPA CRISPR				RNR-RPA CRISPR				RNR-XRT-RPA			
**Dilution**	**RNR C_t**	**~4CP Copies**	**Rep 1**	**Rep 2**	**Rep 3**	**%Positive**	**Rep 1**	**Rep 2**	**Rep 3**	**%Positive**	**Rep 1**	**Rep 2**	**Rep 3**	**%Positive**
**1**	24.52	13600.00	+	+	+	100.00%	NT	NT	NT	-	NT	NT	NT	-
**2**	27.75	1360.00	+	+	+	100.00%	NT	NT	NT	-	NT	NT	NT	-
**3**	31.27	136.00	+	+	+	100.00%	NT	NT	NT	-	NT	NT	NT	-
**4**	32.75	44.80	+	+	+	100.00%	NT	NT	NT	-	NT	NT	NT	-
**5**	34.40	13.60	+	+	+	100.00%	+	+	+	100.00%	-	-	-	0.00%
**6**	36.42	4.53	-	-	+	33.33%	-	-	-	0.00%	-	-	-	0.00%
**7**	37.17	1.36	-	-	-	0.00%	NT	NT	NT	-	NT	NT	NT	-
**8**	38.06	0.45	-	-	-	0.00%	NT	NT	NT	-	NT	NT	NT	-
**NTC**	-	-	-	-	-	-	-	-	-	-	-	-	-	-

			CLas-positive sample 2024-05 (Florida)											
			4CP-RPA CRISPR				RNR-RPA CRISPR				RNR-XRT-RPA			
**Dilution**	**RNR C_t**	**~4CP Copies**	**Rep 1**	**Rep 2**	**Rep 3**	**%Positive**	**Rep 1**	**Rep 2**	**Rep 3**	**%Positive**	**Rep 1**	**Rep 2**	**Rep 3**	**%Positive**
**1**	25.64	1360.00	+	+	+	100.00%	+	+	+	100.00%	+	+	+	100.00%
**2**	29.29	136.00	+	+	+	100.00%	+	+	+	100.00%	+	+	+	100.00%
**3**	33.26	13.60	+	+	+	100.00%	+	+	+	100.00%	+	+	+	100.00%
**4**	34.59	4.53	+	+	+	100.00%	+	+	+	100.00%	+	+	+	100.00%
**5**	36.06	1.36	-	-	-	0.00%	-	-	-	0.00%	-	-	-	0.00%
**6**	37.93	0.45	-	-	-	0.00%	-	-	-	0.00%	-	-	-	0.00%
**NTC**	-	-	-	-	-	-	-	-	-	-	-	-	-	-

NT – not tested

Table S2. Sensitivity data using dilution series tested in the Genie® III.

Dilution	RNR C_t	~4CP Copies	California											
			4CP-RPA CRISPR				RNR-RPA CRISPR				RNR-XRT-RPA			
			Rep 1	Rep 2	Rep 3	%Positive	Rep 1	Rep 2	Rep 3	%Positive	Rep 1	Rep 2	Rep 3	%Positive
1	24.47	13600.00	+	+	+	100.00%	NT	NT	NT	-	NT	NT	NT	-
2	27.74	1360.00	+	+	+	100.00%	NT	NT	NT	-	NT	NT	NT	-
3	31.02	136.00	+	+	+	100.00%	NT	NT	NT	-	NT	NT	NT	-
4	32.69	44.80	+	+	+	100.00%	NT	NT	NT	-	NT	NT	NT	-
5	33.95	13.60	+	+	+	100.00%	+	+	+	100.00%	+	+	+	100.00%
6	36.05	4.53	-	-	+	33.33%	+	-	-	33.33%	+	+	+	100.00%
7	37.47	1.36	-	-	-	0.00%	NT	NT	NT	-	NT	NT	NT	-
8	37.95	0.45	-	-	-	0.00%	NT	NT	NT	-	NT	NT	NT	-
NTC	-	-	-	-	-	-	-	-	-	-	+	+	+	100.00

Dilution	RNR C_t	~4CP Copies	Texas											
			4CP-RPA CRISPR				RNR-RPA CRISPR				RNR-XRT-RPA			
			Rep 1	Rep 2	Rep 3	%Positive	Rep 1	Rep 2	Rep 3	%Positive	Rep 1	Rep 2	Rep 3	%Positive
1	24.52	13600.00	+	+	+	100.00%	NT	NT	NT	-	NT	NT	NT	-
2	27.75	1360.00	+	+	+	100.00%	NT	NT	NT	-	NT	NT	NT	-
3	31.27	136.00	+	+	+	100.00%	NT	NT	NT	-	NT	NT	NT	-
4	32.75	44.80	+	+	+	100.00%	NT	NT	NT	-	NT	NT	NT	-
5	34.40	13.60	+	+	+	100.00%	+	+	+	100.00%	+	+	+	100.00%
6	36.42	4.53	-	+	+	66.67%	+	-	+	66.67%	+	+	+	100.00%
7	37.17	1.36	-	-	-	0.00%	NT	NT	NT	-	NT	NT	NT	-
8	38.06	0.45	-	-	-	0.00%	NT	NT	NT	-	NT	NT	NT	-
NTC	-	-	-	-	-	-	-	-	-	-	+	+	+	100.00

Dilution	RNR C_t	~4CP Copies	CLas-positive sample 2024-05 (Florida)											
			4CP-RPA CRISPR				RNR-RPA CRISPR				RNR-XRT-RPA			
			Rep 1	Rep 2	Rep 3	%Positive	Rep 1	Rep 2	Rep 3	%Positive	Rep 1	Rep 2	Rep 3	%Positive
1	25.64	1360.00	+	+	+	100.00%	+	+	+	100.00%	+	+	+	100.00%
2	29.29	136.00	+	+	+	100.00%	+	+	+	100.00%	+	+	+	100.00%
3	33.26	13.60	+	+	+	100.00%	+	+	+	100.00%	+	+	+	100.00%
4	34.59	4.53	+	+	+	100.00%	+	+	+	100.00%	+	+	+	100.00%
5	36.06	1.36	-	-	-	0.00%	-	-	-	0.00%	-	-	-	0.00%
6	37.93	0.45	-	-	-	0.00%	-	-	-	0.00%	-	-	-	0.00%
NTC	-	-	-	-	-	-	-	-	-	-	-	-	-	-

NT – not tested

Table S3. Known CLas-positive ACP and citrus samples used in this study and corresponding real-time PCR and RPA-CRISPR data.

Sample	Host Type	RNR PCR	4CP RPA-CRISPR
724	ACP	28.52	+
727	ACP	29.02	+
733	ACP	29.62	+
735	ACP	29.62	+
759	ACP	28.82	+
783	ACP	28.02	+
789	ACP	28.72	+
795	ACP	28.52	+
798	ACP	30.02	+
802	ACP	28.12	+
808	ACP	29.62	+
819	ACP	29.02	+
820	ACP	29.32	+
827	ACP	29.42	+
828	ACP	28.22	+
829	ACP	29.72	+
832	ACP	29.72	+
834	ACP	28.92	+
837	ACP	29.52	+
855	ACP	29.72	+
858	ACP	29.42	+
863	ACP	31.22	+
877	ACP	30.72	+
930	ACP	30.82	+
937	ACP	29.52	+
939	ACP	31.42	+
941	ACP	30.32	+
943	ACP	30.82	+
948	ACP	31.52	+
950	ACP	31.34	+
958	ACP	30.62	+
B430-1	Plant	18.43	+
B430-2	Plant	19.31	+

Table S4. Diagnostic samples used in this study and corresponding real-time PCR and RPA-CRISPR data.*

Sample	Host Type	RNR PCR	4CP RPA-CRISPR
2018-01A	Plant	0.00	-
2018-01B	Plant	0.00	-
2018-02A	Plant	37.90	-
2018-02B	Plant	36.32	+
2018-03A	Plant	36.11	+
2018-03B	Plant	37.38	+
2018-04	Plant	37.77	+/-
2018-05A	Plant	37.52	-
2018-05B	Plant	0.00	-
2018-06A	Plant	36.64	-
2018-06B	Plant	36.21	-
2019-01	Plant	23.94	+
2022-01A	Plant	22.47	+
2022-01B	Plant	23.51	+
2022-01C	Plant	23.10	+
2022-02A	Plant	21.76	+
2022-02B	Plant	21.30	+
2022-03A	Plant	0.00	-
2022-03B	Plant	0.00	-
2023-01	Plant	20.97	+
2023-02A	Plant	17.81	+
2023-02B	Plant	17.58	+
2023-03A	Plant	17.38	+
2023-03B	Plant	17.35	+
2023-04	Plant	22.01	+
2023-05	Plant	22.33	+
2024-01	Plant	19.05	+
2024-02	Plant	19.55	+
2024-03	Plant	20.50	+
2024-04	Plant	20.11	+
2024-05	Plant	19.20	+
2024-06	Plant	22.70	+
2024-07	Plant	22.40	+
2024-08	Plant	24.01	+
2024-09	Plant	0.00	-
2024-10	ACP	26.40	+
2025-01	Plant	21.55	+
2025-02	ACP	23.52	+
2025-03	Plant	28.48	+
2025-04	Plant	25.78	+
2025-05	ACP	25.38	+
2025-06	Plant	22.93	+
2025-07	Plant	24.99	+
2025-08	Plant	21.64	+

*Highlighted samples = low-titer/high-C_t (>36) samples from 2018, many reported as inconclusive; Green = positive by RNR real-time PCR and 4CP RPA-CRISPR; Light green = positive by RNR real-time PCR and 4CP RPA-CRISPR, but with less than 100% positivity for 4CP RPA-CRISPR (possible DNA degradation); Red = positive by RNR real-time PCR and negative by 4CP RPA-CRISPR (possible DNA degradation).

Table S5a. RPA-CRISPR Primer Mix (pMix)

Primer & Probe	Volume (µl)	Working Conc. (µM)
F (100 µM)	50	5
R (100 µM)	50	5
MG Water	900	---
Total	1,000	N/A

Table S5b. Component A.[a]

Reagent	1 Reaction (µl)	Concentration	Final Concentration
10X NEBuffer 2.1[b]	2.5	4.17X	0.83X
MG Water	0.875	---	---
ssDNA Reporter (10 µM)	0.625	1.042 µM	0.208 µM
MgOAC (280 mM)[c]	1.5	70 mM	14 mM
crRNA (2.5 µM)	0.25	0.1042 µM	0.021 µM
Cas12a (Cpf1) (2.5 µM)[b]	0.25	0.1042 µM	0.021 µM
**Total Reaction**	**6.0**	---	---

[a]Component A is heated to 37°C for 10 min and then stored on ice for use.
[b]These reagents are provided with the EnGen® Lba Cas12a (Cpf1) 100 µM (2,000 pmol) (New England Biolabs, Ipswich, MA).
[c]This reagent is provided with the TwistAmp® Basic lyophilized pellets (TwistDx™ Ltd., Cambridge, UK).

Table S5c. Component B.

Reagent	1 Reaction (µl)	Concentration	Final Concentration
Rehydration Buffer[a]	29.5	---	---
pMix (5 µM each)	4.8	0.539 µM each	0.396 µM each
MG water	10.2	---	---
**Total Reaction**	**44.5**	---	---

[a]This reagent is provided with the TwistAmp® Basic lyophilized pellets (TwistDx™ Ltd., Cambridge, UK).

Table S6. Nucleotide sequence of the synthetic gBlocks™ positive control for '*Candidatus* Liberibacter asiaticus' detection using the RPA-CRISPR-Cas-based assay.

4CP-250
ACAGTAATTGTAAACTGACTAAACCAATTAGTTATAACATTACGTGCAAGACTTTT<br>GGCTATAGGCATGACATTCTCGTCAGAATTAATTTCATAATATTTAGATGGATCAC<br>TTGCACATTGTCTTAAAGTATATCGCGTATCTTGATCCGGAGAAAATCCTACAGAA<br>AATATGGTAATTGCATCTCTTCCAGTTCTATGACGCTTATGCATGTAATGGCAGTC<br>TTGCAGAACCTTTTCTATTTTTTCTT
